# Ethical problems in intensive care unit admission and discharge decisions: a qualitative study among physicians and nurses in the Netherlands

**DOI:** 10.1186/s12910-015-0001-4

**Published:** 2015-02-26

**Authors:** Anke JM Oerlemans, Nelleke van Sluisveld, Eric SJ van Leeuwen, Hub Wollersheim, Wim JM Dekkers, Marieke Zegers

**Affiliations:** Scientific Institute for Quality of Healthcare (IQ healthcare), Radboud University Medical Center, PO Box 9101, 6500 HB Nijmegen, the Netherlands; Department of Intensive Care Medicine, Radboud University Medical Center, PO Box 9101, 6500 HB Nijmegen, the Netherlands

**Keywords:** Critical Care, Ethics, Intensive Care Units, Qualitative Research, Rationing, Resource Allocation

## Abstract

**Background:**

There have been few empirical studies into what non-medical factors influence physicians and nurses when deciding about admission and discharge of ICU patients. Information about the attitudes of healthcare professionals about this process can be used to improve decision-making about resource allocation in intensive care. To provide insight into ethical problems that influence the ICU admission and discharge process, we aimed to identify and explore ethical dilemmas healthcare professionals are faced with.

**Methods:**

This was an explorative, descriptive study using qualitative methods (individual and focus group interviews). We conducted 19 individual interviews and 4 focus group interviews with nurses and physicians working in the ICU or the general ward of 10 Dutch hospitals.

**Results:**

The ethical problems in the context of ICU admission and discharge can be divided into problems concerning full bed occupancy and problems related to treatment decisions.

The gap between the high level of care the ICU can provide and the lower care level in the general ward sometimes leads to mutual misunderstandings. Our results indicate that when professionals of different wards feel there is a collective responsibility and effort to solve a problem, this helps to prevent or alleviate moral distress.

ICU patients’ wishes are often unknown, causing healthcare professionals to err on the side of more treatment. Additionally, the highly technological nature of intensive care appears to encourage over-treatment.

**Conclusions:**

It is important for ICUs and general wards to communicate and cooperate well, since there is a mutual dependency for optimal patient flow between the different departments. Interventions that improve the understanding and cooperation between these wards may help mitigate ethical problems.

The nature of the ICU environment makes it important for healthcare professionals to be aware of the risk of over-treatment, reflect on why they do what they do, and be mindful of a possible negative impact of over-treatment on their patients. Early discussion of a patient’s wishes with regard to treatment options is important in preventing over-treatment.

## Background

The intensive care unit (ICU) is a high pressure environment, where expensive care is delivered by highly qualified personnel to patients suffering from potentially life-threatening diseases. Bed availability is limited, making high patient throughput important. This throughput is dependent on the admission of new patients, and discharge to general wards of those whose ICU care requirement is supposed to have ended. Financial pressure from society as well as higher management is increasing. Critical care services represent an increasing proportion of total hospital costs, up from 8% in 1980 to 20% in 2006 in the United States [1]. In the Netherlands, the costs of ICU departments have been estimated to represent approximately 20% of the total hospital budget [2]. The limited number of ICU beds as well as the pressure of ICU care on the total hospital budget, necessitates optimal use of ICU beds and patient flow from emergency room, operating theatre and general ward to ICU and vice versa.

The ICU is an ethically charged environment: life and death decisions are made daily, in acute, highly emotional situations that often involve legally incompetent patients and their family. Decisions to admit or discharge a patient are often not merely medical decisions. Non-medical aspects, such as pressure from managers or patients, may play a role in the decision-making process [3-5]. Therefore, the ideal of decision-making based on objective medical criteria can be very difficult to achieve.

Particularly when medical criteria alone are insufficient in deciding what is the right thing to do, healthcare professionals can be faced with an ethical dilemma; a conflict of values can occur which makes every possible decision less than optimal on moral grounds. For instance, deciding whether to discharge a patient not quite ready for the general ward to create bed space for a gravely ill patient in need of intensive care [6-8].

There have been few empirical studies into what non-medical factors influence physicians and nurses when deciding about admission and discharge of ICU patients [3-5,9]. These studies were predominantly based on quantitative questionnaire research and focused solely on the ICU perspective. They found considerable variation between countries and individual practitioners with respect to the factors taken into account. In addition, they showed that pressure from supervisors or managers, referring physicians, family or patients may influence decisions to admit or discharge ICU patients [3-5]. These factors may cause the healthcare professionals involved to experience moral distress: stress that “occurs when one knows the right thing to do, but institutional or other constraints make it difficult to pursue the desired course of action” [10-12].

So far, little is known about the views of healthcare professionals involved in the admission and discharge process. There is a dearth of research that includes all relevant perspectives – physicians and nurses from both the ICU and the general ward – and uses qualitative methods to explore these views in depth. Information about the attitudes of healthcare professionals about this process can be used to improve decision-making about resource allocation in intensive care. To provide insight into ethical problems that influence the ICU admission and discharge process, we aim to identify and explore ethical dilemmas healthcare professionals are faced with.

## Methods

### Design and setting

This is a descriptive, explorative study in which qualitative methods (individual and focus group interviews) are used to identify and explore ethical dilemmas. Semi-structured face to face interviews are useful to explore sensitive topics in-depth [13]. In the subsequent focus groups, the group dynamic and interaction among participants helps to further explore and clarify participants’ views [13].

We included Dutch hospital physicians and nurses, working in either the ICU department, or a general ward regularly admitting patients from the ICU.

### Individual interviews

Before the start of the study, contact was established by telephone with ICU physicians in six hospitals: two general, two teaching, and two academic hospitals. Through these six hospital contacts, physicians and nurses were recruited for face to face interviews. Inclusion criterion was involvement in (post-)ICU patient care; working as a physician or nurse in either the ICU, or in a general ward regularly admitting post-ICU patients. The prospective participants were informed by email about the objective of the study, and were invited to participate. The interviews took place at the participants’ place of work. The amount of interviews depended on the point of saturation, in other words when no new information could be identified in the interviews [13]. An interview guide with open-ended questions was developed and pilot tested. For an example of the ICU nurse interview guide, see “Example of interview guide”.

All interviews were held between April and December of 2012. They were conducted by a trained and experienced interviewer (AO), in the presence of one other researcher (NvS). Audio of the interviews was recorded and subsequently transcribed verbatim.

### Example of interview guide

Interview guide for ICU nurse interviews.

- What ethical dilemmas surrounding ICU admission and discharge do you face in your daily work?
- Do you ever disagree with the decision to admit a patient to the ICU? Please give an example.
- Do you ever disagree with the decision to discharge an ICU patient? Please give an example.
- Could you give an example of a situation in which problems arose regarding the admission of an ICU patient?
- Could you give an example of a situation in which problems arose regarding the discharge of an ICU patient?
- What happens when all ICU beds are occupied and the ICU receives a request for an ICU bed?
- Could you give an example of this happening?
- How was this acted upon?

The interview guides for the other types of stakeholder interviews are available on request.

### Focus group interviews

To explore the themes and dilemmas identified in the individual interviews more in depth, we conducted four focus group interviews with: (1) ICU physicians, (2) ICU nurses, (3) general ward physicians, and (4) general ward nurses. A focus group interview guide was designed around two or three fictional cases, all of which were checked for medico-technical accuracy by three physicians (for an example, see “Example of a fictional case used in the focus groups”). These cases were compilations of dilemmas described by interview participants and/or case studies found in the literature [14-17].

Recruitment for the focus group interviews took place through snowball sampling: initially, the ICUs and general wards of the six initial hospitals were contacted and through these contacts, physicians and nurses of relevant wards in other hospitals were contacted and invited. The participants were sent the case descriptions by email and were asked to read them in advance. The focus group interviews were led by a moderator (WD for focus groups 1 and 2, AO for focus groups 3 and 4), respectively three (NvS, MZ, AO) and two (NvS, MZ) other researchers were present for the discussions, both to observe as well as to assist the moderator. Each focus group interview commenced by explaining the goal of the meeting, introducing the researchers, and introducing the focus group participants.

The focus group meetings took place in January of 2013. Audio of the interview was recorded, and a note taker was present at the meetings. The interviews were transcribed verbatim. The transcript of the focus group interviews was sent to the participants for corrections and additional comments.

### Example of a fictional case used in the focus groups (for general ward physicians and general ward nurses)

Mr. Anouh, 68 years old, has been on hemodialysis for a number of years due to renal failure. He was admitted to the Nephrology Department with a staphylococcal sepsis, where they are experiencing problems in keeping his blood pressure up. He has been given lots of fluids, but his blood pressure remains low. Then, he develops a watershed infarction (stroke). Sometime that afternoon, the ward doctor calls the ICU for a consult. The ICU physician on duty indicates that he wants to admit mr. Anouh, but does not have a bed available at the moment – “we don’t have another solution right now, just keep filling him,” is his message. It turns 5pm, 6pm, 7pm, and mr. Anouh is still at the nephrology ward, where his condition keeps deteriorating. In the meantime his family has arrived. They are very upset about the state of affairs, since his nurse had told them hours ago that he would be admitted to the ICU. Mr. Anouh’s nurse feels very powerless too.

### Ethical approval

Ethical approval was sought from the Research Ethics Committee of the Radboud University Nijmegen Medical Centre (registration number: 2011/483); the committee judged that ethical approval was not required under Dutch National Law. All participants received written information about the project and its aims, and were subsequently invited to participate. We stressed that participation in this study was voluntary and withdrawal from the study was possible at any time. The anonymity of participants and institutions was maintained in the interview transcripts.

### Analysis

The interview and focus group transcripts were coded using ATLAS.ti 6.2 (developer: ATLAS.ti Scientific Software Development GmbH). The analysis was conducted using a grounded theory approach, in which the codes and codebook emerge from the data (as opposed to previously formulated hypotheses which are “tested” against qualitative data) [18,19]. The first five individual interviews were coded by AO, NvS and MZ, after which any discrepancies were discussed until consensus was reached. A double analysis (AO and MZ) and subsequent discussion was also performed for the first focus group interview transcript. All other transcripts were coded by one researcher (AO). We used the COREQ guideline for qualitative research for both design and analysis [20]. Our study adhered to BioMed Central’s modified RATS guidelines.

## Results

### Study population

We conducted 19 semi-structured individual interviews with ICU physicians, ICU nurses, general ward physicians, and general ward nurses (for participant characteristics, see Table 1). The interviews took between 30 and 120 minutes. All invited agreed to participate, except for one ICU physician who declined for scheduling reasons.

**Table 1:** **Characteristics of interview and focus group participants**

	**Interviews (n = 19)**	**Focus groups (n = 25)**
**Job title**		
ICU physician (%)	7 (37)	5 (20)
ICU nurse (%)	6 (32)	7 (28)
Ward physician (%)	3 (16)	5 (20)
Ward nurse (%)	3 (16)	8 (32)
**Male (%)**	11 (58)	8 (32)
**Hospital type**		
General (%)	6 (32)	5 (20)
Teaching (%)	7 (37)	10 (40)
Academic (%)	6 (32)	10 (40)
**Years experience in current specialty**		
<5 years (%)	7 (37)	5 (20)
5 – 10 years (%)	4 (21)	7 (28)
>10 years (%)	8 (42)	13 (52)

We conducted four focus group interviews with ICU physicians, ICU nurses, general ward physicians, and general ward nurses (Table 1). The focus group interviews took between 60 and 90 minutes. Seventeen ICU physicians were invited, 5 were present at the focus group interview. Thirty-six general ward physicians were invited, 5 of whom participated in the interview. Twenty-five ICU nurses were invited, 7 of whom took part in the interview. Twenty-five general ward nurses were invited, 8 of whom participated in the focus group.

### Ethical problems

The individual and focus group interviews showed that in the context of the ICU admission and discharge process, ethical problems arise at different points in time: (A) when (deciding about) admitting a patient to the ICU from the emergency room, operating theater or a general ward, (B) during a patient’s stay in the ICU, (C) when (deciding about) discharging a patient from the ICU. We will now go through these phases, and elaborate on the different ethical problems healthcare professionals encounter in their work.

#### Phase A: Admission to the ICU

For quotations related to phase A, see Table 2.

**Table 2:** **Ethical problems related to ICU admission**

**Problem**	**Participant**	**Representative quotes**
Delayed/refused admission	General ward nurse	*“Look, you see a patient deteriorate and be sad and in fear and pain, and at a certain point you can’t really do much more than what the doctor says you should do and what you know you should so at that time. Of course, initially that’s the most important thing, but at a certain point you can’t do more than execute the doctor’s orders and keep the patient as stable as possible, but the capabilities of a general ward are pretty limited, you know? And then it’s just waiting for what a doctor decides and sometimes that’s..that takes a very long time”.*
	General ward physician	*“If we* [the general ward physician and the consulting ICU physician, AO] *agree that the patient in question is actually an ICU patient, then I think it should be a shared responsibility. If our own ICU doesn’t have a bed, then another bed in a different place needs to be found. And then it’s not like ‘I just don’t have a bed’.”*
Need to transfer a patient to a different hospital (according to guideline)	ICU physician	*“In the beginning, I had a lot of problems with it* [the guideline on an admission request in case of full bed occupancy, AO]*, the way it was drawn up. It went completely against my own way of thinking. I took the risks as a starting point. Which patient can you help the most here, who will suffer most from not being admitted at that moment?”*
	ICU physician	*“There was an unspoken agreement among ICU physicians that the patient with the lowest risk went. Always. The lowest transport risk is the one to go. The guideline interfered with that concept”.*
	ICU physician	*“This is an intrinsic error in the guideline. I think we all feel very strongly that you should act on the basis of clinical insight and weighing of risks”.*
Difference of opinion about the start of ICU treatment	General ward physician	*“I notice that the longer I’ve worked here, I kind of got..for me it’s kind of a slippery slope, because they’re not really well-defined terms you know, what futility is. I think futility is a very subjective concept and what you consider futile can be very meaningful for me, very valuable, just, that’s the way it is for such a patient too. In the beginning I was more straightforward, and now my thinking is much more nuanced and I can more easily go along with family in those cases than a couple of years ago”.*
	General ward physician	*“To a large extent it is our interpretation of such an existence or of that quality of life, of which we think - well, is that worth the effort? Even though at such an acute time, that could be completely different for the family or the patient. I have a couple of patients that, well, literally are unable to do anything but lie in bed all day without consciousness but the family still considers it to be very meaningful”.*
	ICU physician	*“I think we sometimes admit people we shouldn’t admit, and I think that sometimes we can say in advance that we shouldn’t have let this patient go to the ICU, but we’re too afraid that we judge things too negatively and we do it anyway, but with the result that we treat the patient for too long”.*
	ICU physician	*“I’ve come to an age where I’ve become careful. I’ve been wrong too many times. You can only stop once”.*
Decision to admit/treat was based on inaccurate/incomplete information	ICU physician	*“The worst, I think, is when a patient is admitted who was resuscitated in the general ward and the family comes in a short time later and says - ‘daddy wouldn’t have wanted this’. Then real lines were crossed, invasive medical acts were performed based on misinformation. Well, I think that’s a shame”.*
	ICU physician	*“Look, when it’s very difficult to keep a patient stable, it’s simple. Then you just pull out the tube, give a little morphine: done, you know? But if the patient becomes nice and stable, well, then you have a very difficult problem of course”.*

##### Delayed or refused admission to the ICU

As our participants indicated, ICU bed pressure causes problems for general wards (including the emergency room) with a patient in need of ICU care: ICU admission is delayed or sometimes even refused, and elective surgeries are cancelled because no post-surgery ICU bed is available. In addition to possible negative consequences to a patient’s health, this leads to frustration for both the patient and his family, as well as for nurses and physicians in the general ward in question. At the heart of this moral distress is the desire to provide the best care possible, but being unable to do so, often for reasons beyond the caregiver’s control. The aforementioned fictional case of Mr. Anouh (See “Example of a fictional case used in the focus groups”) is an example of such a situation. As the interviews made clear, these situations are especially difficult for the general ward nurses. As several general ward nurses described, the combination of being in close proximity to the patient, but not being the one making the decisions can lead to a feeling of tremendous powerlessness. For the general ward physicians interviewed, too, these situations can be difficult, especially when they have the impression that they carry sole responsibility for the well-being of this patient. The degree to which they feel the ICU physician takes his or her responsibility to try and solve the problem, greatly influences the distress general ward physicians experience, as they indicated in the interviews.

Participants highlighted that because of the mutual dependency of the ICU and the general ward, collaboration between the healthcare professionals in both wards is important to ensure optimal patient flow and care. The interviews made clear that general ward personnel perceive the ICU and its personnel as different from the “regular” wards. This is due in part to the physical separation of the ICU from the rest of the hospital (separate wing, closed doors, need to ring the bell before entering etc.), and partly to the perceived psychological distance between the different wards . Terms such as “arrogant”, “ivory tower”, and “island” were used often, although participants mentioned that the situation has improved over time, and the behavior varies between persons. In particular general ward nurses described they sometimes perceived a barrier when having to call the ICU about a patient, or asking ICU personnel for help with certain medical interventions.

##### Need to transfer a patient to a different hospital (according to guideline)

All participants recognized the situation of full ICU bed occupancy, but the frequency with which it happens and the degree to which they experience it as a problem varies. In some cases, it means that a patient in need of ICU care – either from one of the general wards or brought in through the emergency room – is stabilized and subsequently transported to be cared for in a different ICU in another hospital, meanwhile being at risk through delay of (intensive) care and the transport itself. Sometimes, a stable patient in the ICU is transferred to an ICU elsewhere, often because the new patient is deemed too unstable to withstand care delay and transport. In the Netherlands, a guideline pertaining to this situation (“Admission request in case of full ICU bed occupancy” of the Dutch Society for Intensive Care) prescribes that in principle, no patients already admitted to the ICU should be transferred to make room for a new admission, since there is a treatment contract between admitted patients and their physician/the hospital, that cannot be terminated unilaterally [21]. The vast majority of the ICU physicians admitted they did not use the guideline in daily practice. The importance of an existing treatment relationship in deciding who to transfer was disputed by several physicians. They indicated that they preferred (and still used) a risk-based approach; the patient with the lowest transport risk would be the one to go.

##### Difference of opinion about the start of ICU treatment

Questions surrounding the futility of treatment were one of the most frequently mentioned sources of ethical dilemmas, especially among ICU physicians and nurses. Although there is often consensus surrounding the decision to admit a patient to the ICU and start intensive treatment, in some cases those involved disagree on the right thing to do for the patient in question – be it ICU personnel, general ward personnel, family or the patient him- or herself.

According to our participants, the expected quality of life after hospital discharge appeared to be the main deciding factor in dilemmas about the possible futility of ICU treatment. However, many participants recognized the subjectivity of these quality of life predictions. When in doubt, therefore, physicians tended to err on the side of treatment. Participants indicated that sometimes, a patient is treated longer or more aggressively than the patient would have wanted. In those cases a patient’s wishes are often unknown to the treating physician, either because they have not been discussed, or because they were not adequately recorded in the patient’s records. ICU physicians expressed frustration that they are regularly confronted with elderly patients in poor condition who spent a substantial amount of time in a general ward, without general physicians having discussed their treatment wishes with them.

##### Decision to treat was based on inaccurate/incomplete information

As many respondents described, inherent to critical care medicine is the need to make decisions in acute situations: a critically ill patient is brought in to the emergency room, or a general ward is suddenly faced with a patient in cardiac arrest. This context sometimes leads to decisions based on inaccurate information, such as the decision to resuscitate someone who is later found to have a do-not-resuscitate order in place. These spur-of-the-moment decisions can then lead to complex dilemmas, especially when a patient remains in stable but serious condition after resuscitation, with the patient’s family indicating the patient in question would not have wanted it that way.

#### Phase B: In the ICU

For quotations related to phase B, see Table 3.

**Table 3:** **Ethical problems in the ICU**

**Problem**	**Participant**	**Representative quotes**
Expansion of treatment indications	ICU physician	*“There are a number of cases we wouldn’t have touched in the past. We wouldn’t even have been asked to consult on them. Or when we would’ve been asked, then it would always be like: ‘well, you’re not going to start this, but can you just help us with the decision not to go to the ICU with this’. But now..you see it shift because we’ve seen patients come out of a situation like that, who we’ve then given a period of good time afterward”.*
	ICU nurse	*“The very serious COPD patients..those weren’t admitted about 10 years ago because they just thought - those aren’t going to make it. And now we still know that the odds of them making it are slim, and they are going to need a rehabilitation course of a year to survive. Well, what did we achieve then?”*
Difference of opinion about the stopping of treatment	ICU physician	On the difference between physicians and nurses:
		*“Their contact with the patient is much more intense. They’re at the bedside daily, experience the patient daily, how they are feeling, what mood they’re in, those kinds of things. They suffer with them, they feel what the patient feels”.*
	ICU nurse	*“As a nurse you see the patient more often, or you speak with the family more often. As a doctor you only see the patient when you come into the room and therefore, as a nurse, you have more feelings about..well..what you’re doing, whether you agree with it. And I think as a nurse you more often feel like, well, maybe we should stop this. Because a doctor is very much like – you’re there to make the patient better and, they don’t see the patient for very long. In any case, I can’t think of an example in which the doctor was like – ‘well, let’s stop this’, and the nurse didn’t agree with it”.*
	ICU nurse	*“I remember a case of a pretty young guy, not yet fifty, who had had a motorcycle accident and had both legs amputated above the knee, as well as part of his arm. So he was lying in bed with one complete arm plus internal injuries and then you think, just let someone like that..because he’ll get lots of complications. With that case we* [the ICU nurses; AO] *were like ‘you shouldn’t do this’. Eventually the ICU physician pushed through. To this day, once every year that guy comes by to thank everyone, that they kept on and treated him. Then I’m like - here I am with my big mouth”.*
	ICU physician	On individual differences among ICU physicians:
		*“Among our staff, and I think that’s the case everywhere, there is a difference between people who are quicker to stop treatment and people who’d rather never stop, and the grey zone in the middle. Those people with the scythe who want to abstain everyone at every turn, they’re too fast. But the people who never want to stop because they think ‘well, you can never be sure, I won’t make that decision, I don’t dare to take on that responsibility’, well, they go on too long with people that really should have passed away ages ago. And the truth lies somewhere in the middle, but that’s why it’s good to discuss a decision like that during a multidisciplinary meeting, so that all the different perspectives can give their response, and that is how it happens.”*
	ICU physician	On the reluctance of some physicians to abstain a patient from treatment:
		*“That is driving up healthcare costs, giving the family false hope, causing the nursing staff to become demotivated because they already know it’s not going to work. But it takes guts and that’s a problem. It takes guts to decide this, it takes guts to go and talk to the family and say it out loud, it takes guts to explain to your colleagues that you’re not going to continue. Well, for me it’s not that hard, but it is a difficult part of our profession, with which some of my colleagues clearly have more trouble”.*
Patient is stable after withdrawing life-sustaining treatment	ICU physician	*“The patient was expected to die soon, but that didn’t happen. Then you have to, then you transfer a patient to the ward, who’ll only go there to die. That is..that was difficult for a while. But we do need that bed”.*
	ICU nurse	*“We had a case in which the doctor said - ‘okay, we’re going to transfer him to the general ward, and then a new admission will come in his place’. No way, we’re not going to do that. Then they’ll just have to transfer out someone else. But those are..we really fought about that one, you know”.*
Patient is stable as long as ICU care is given	ICU nurse	*“It’s specifically the group that kind of slips through all the cracks, a patient who starts to breathe on his own, those are the difficult categories. No one will be able to make a decision about that. Like, he breathes on his own, let’s wait and see. And ultimately it becomes days, weeks”.*
	ICU physician	*“We create this type of patient because we can do so much […] It’s a good thing to have these patients now and again. It means the aggressiveness of your treatment is high. It means that you don’t deprive a great number of other patients. But this is the risk of being very aggressive, or going very far in your treatment”.*

##### Expansion of treatment indications

Several healthcare professionals interviewed, mainly from the ICU, perceived a shift in what conditions are treated; they witnessed an expansion of treatment indications for certain interventions. When introduced, a certain treatment was only applied in a very specific group of patients (for example, mechanical ventilation for patients with a moderate case of COPD), but over the years (especially when the treatment looked to be beneficial in many patients) the types and severity of illness it is used to treat has broadened. On the one hand, this development was considered a positive one – a sign of scientific and technical progress. Others, specifically ICU nurses, were critical of this development, indicating that it led to prolonged treatment that they did not consider beneficial to the patients in question.

##### Difference of opinion about the stopping of treatment

Similar to the differences of opinion surrounding the admission of a patient to the ICU, there can be a difference of opinion about whether to continue or withdraw life-sustaining treatment of a patient already in ICU, whether it be between family and hospital staff, between physicians and nurses, or between individual healthcare professionals. When our participants describe these situations, the difference in perspective between nurses and physicians become very apparent. Where physicians have a “cure” perspective and have a decision-making role, nurses work from a “care” perspective and spend more time at the patient’s bedside, in direct contact with the patient and their family. Without exception, our respondents indicated that generally, when there is a difference of opinion about the futility of continuing treatment it is the nursing staff that is in favor of withdrawing treatment while physicians want to continue.

##### Patient is stable after withdrawing life-sustaining treatment

As our participants described, when the decision is made to withdraw life-sustaining treatment in the ICU, the expectation is that the patient will die shortly. In some cases however, the patient in question remains in stable condition, and although he/she is still expected to die in the near future, it is impossible to predict exactly when. Strictly speaking, such a patient is no longer in need of ICU care and is occupying a bed that other patients are in urgent need of. Here, we again noticed a clear difference between those in the decision-making role, ICU physicians, and those in the care role, ICU nurses. Where nurses are focused on the needs of the individual patient and their family, physicians are also responsible for optimizing the patient flow into and out of the unit.

##### Patient is stable as long as ICU care is given

Our participants indicated that with the increased technological possibilities, the ICU is able to sustain increasingly sicker patients for longer periods of time. This leads to cases of patients that are in stable condition in the ICU, sometimes even awake and able to communicate, as long as intensive treatment is provided. There is no chance of these patients ever being able to leave the ICU, but as long as life-sustaining treatment is not withdrawn, they will carry on living. Several participants described these situations as emotionally draining, especially when patients are able to communicate.

#### Phase C: Discharge from ICU

For quotations related to phase C, see Table 4.

**Table 4:** **Ethical problems related to ICU discharge**

**Problem**	**Participant**	**Representative quotes**
Premature/suboptimal discharge	General ward nurse	*“We as a general ward think beforehand - ‘we cannot give that care’ and the ICU thinks - ’that should absolutely be possible’. I do get it from an ICU point of view, but you just don’t realize what it sometimes means at a general ward. Of course we can give noradrenalin, we know how that works, and we can give certain medications and whatever, but during the nightshift I have fifteen other patients and I can’t be by that bedside every ten minutes. That’s the problem. That’s something we talk about, argue about quite regularly”.*
	General ward nurse	*“We have those borderline cases where you think - we can do it, but it’s almost impossible at our ward, because we can’t check up on those people that often. And we don’t have monitoring, so it’s not like an alarm sounds when things suddenly turn south. And you just have a number of other patients, that is sometimes the problem. It’s not really a matter of being able or allowed to do something, but you just can’t handle it because you have so many other patients and then it’s just irresponsible to have them lie in the ward without monitoring”.*
	ICU nurse	*“Medically speaking the patient is actually well enough to go to the general or the medium care ward, but those wards say: ‘guys, we can’t handle that one. It’s just impossible!’ And sometimes that’s accepted and usually it isn’t. Usually it’s like - ’well, not my problem. Needs to be admitted anyway’.”*

##### Premature or suboptimal ICU discharge

From the interviews it becomes clear that the gap between the ICU and the general ward is often substantial, resulting in suboptimal care for post-ICU patients and mutual misunderstandings and irritation between ICU and general ward. In the view of general ward personnel, the ICU staff sometimes overestimates the technical capabilities of the general ward, the technical skills of the ward personnel, and the amount of time and attention the ward personnel is able to give each patient. In those cases, there is no absolute lack of beds in the general ward, but a relative lack of care capacity in relation to the existing care burden. However, when there is pressure on the beds in the ICU and someone is (almost) ready to be discharged, the ICU pressures the general wards into admitting a post-ICU patient.

## Discussion

In this study we identified ethical problems at three different time points during the ICU admission and discharge process: surrounding ICU admission, during ICU stay and surrounding ICU discharge. They can roughly be divided into two categories: those related to full bed occupancy, and those having to do with treatment decisions. Ethical problems connected to full bed occupancy have in common the weighing of interests (and risks and benefits) of two (or more) patients against each other: delay of ICU care for patient A means that patient B benefits from the care he needs, or a transport risk and the inconvenience of a different hospital for patient C mean that patient D can benefit from timely and adequate care. Ultimately, the main dilemma healthcare professionals face is the inability to provide the best care for all patients, and the necessity to choose the best possible alternative.

The second cluster of ethical problems is related to treatment decisions, more specifically the decision whether to start or to stop ICU treatment. In these dilemmas the central question is what is best for an individual patient – i.e. weighing of the risks and benefits of different alternatives for the patient in question.

The different nature of the role of nurses and physicians in the patient’s care leads to differences in how they perceive and deal with ethical problems. Where physicians predominantly act in a cure role and carry decision-making responsibility when it comes to admitting and discharging patients, nurses work from a care perspective and do not have this responsibility. As we saw in our study, this can lead to a feeling of powerlessness when confronted with an ethical problem nurses cannot “solve” themselves, because they are in close proximity to the patient and his or her family. In situations of doubt or dissension about the start or continuation of treatment, nurses’ physical and emotional proximity to patients can provide valuable information about the burden of treatment on a patient or their family, and may provide a counterweight to over-treatment tendencies. But in turn, their continuous exposure to patients’ suffering may also lead them to underestimate the chances a patient has of a meaningful recovery.

In several of the ethical problems identified, the concept of medical futility is of relevance. Its exact definition is a point of contention, but one definition commonly used distinguishes three types of medical futility: a treatment can be ineffective, disproportionate, or undesirable [22-24]. As the literature describes, a judgment of the ineffectiveness of treatments is made by physicians. Whether the benefits of treatment are proportionate to its burden should be decided by physician and patient together, since they can assess whether the treatment in question is a reasonable means to reach their goal. Finally, whether the goal of treatment is reasonable is determined by the patient, since this implies a judgment of the value of the life of a patient [23,24]. This seems a clear distinction, but several participants indicate that in practice, this distinction is more difficult to make. They indicated that a judgment of ineffectiveness is hardly ever truly “just” a medical decision.

When describing the ethical dilemmas related to treatment decisions, many participants – especially nurses – address the concern that patients are often treated for too long, or too intensively. The nature of intensive care appears to encourage over-treatment (rather than under-treatment), through two mechanisms: the technological imperative and anticipated decision regret. As we saw in our results, medical technology often creates its own desirability; its indications are expanded through a mechanism of “what is possible should be done”, which is also known as the technological imperative in medicine [25-29]. The ICU environment is especially conducive to this imperative; compared to general wards, there is already a strong focus on highly advanced technology. Because of their illness, patients are often unable to make their wishes known and families (acting as patient proxies) are often unsure about a patient’s exact wishes in the given situation, causing healthcare professionals to err on the side of more treatment. In addition, several of the ethical problems described touch on the notion of anticipated decision regret: the fact that people tend to use the concern they will later regret not having intervened as a motivation for intervention (in this case, deciding to (continue to) treat) [30,31]. The nature of the ICU environment makes it important for healthcare professionals to be aware that these mechanisms are in play in their daily work, reflect on why they do what they do, and be mindful of a possible negative impact of over-treatment on their patients. Early discussion of a patient’s wishes with regard to treatment options is important in preventing unwanted over-treatment. Research into one manner of giving shape to the registration of treatment wishes, advance care directives, suggests that uptake of this method among the general public is low [32-34]. More research into the optimal approach of discussing and registering treatment limitations at an early stage is needed.

What became clear from our study was the difference between the ICU and the general ward, and its role in the emergence and mitigation of ethical problems. Care in the ICU is more highly technological and more intensive than in the general ward – ICU personnel are more technically skilled than nurses in the general ward. In addition, the nurse to patient ratio in the ICU is much higher than in the general ward, meaning that a general ward nurse will on average spend less time at her patients’ bedside than an ICU nurse. Sometimes, the gap between the high level of care the ICU can provide and the lower care level in the general ward leads to mutual misunderstandings: for instance, the ICU staff overestimates the nursing care the general ward can provide, and prematurely discharges patients, substantially increasing the care burden at the general ward. There are several solutions used in practice to bridge the gap between the ICU and general ward and to respond to deteriorating post-ICU patients, such as liaison nurses who coordinate the handover of ICU patients to general wards and who support general ward professionals caring for post-ICU patients who still have complex needs, and outreach teams that provide follow-up to patients recently discharged from the ICU [35]. However, more research into the effectiveness of these interventions is needed.

Our results indicate that when professionals of different wards feel there is a collective effort to solve a problem, a shared responsibility towards achieving the best possible care for the patient(s) in question, and some consideration for the limitations of another ward, this helps to prevent or alleviate moral distress. Interventions that improve the understanding and cooperation between these wards are available, such as short internships, case discussions of suboptimal handovers between general ward and ICU (and vice versa), and structured feedback methods [36-41]. It is important for ICU and general ward to cooperate well, since they are cogs in the same machine: there is a mutual dependency for optimal patient flow between the different departments.

### Study limitations

Our study had several limitations. Any qualitative study carries the risk of eliciting false, socially desirable responses from the interviewees, especially when inquiring after topics in the field of ethics. By asking the participants to describe examples of problems they themselves experienced, we hope to have diminished this risk. The absolute number of participants in our study was relatively small. However, when considering the labor-intensiveness of qualitative research and the suggested number of interviews in the literature, the number of interview and focus group participants was more than required [42]. Additionally, by selecting different types of professionals, from different types of hospitals and different wards, we included a breadth of perspectives, increasing the generalizability of our research.

## Conclusions

The nature of the ICU environment makes it important for healthcare professionals to be aware of the risk of over-treatment, reflect on why they do what they do, and be mindful of a possible negative impact of over-treatment on their patients. Early discussion of a patient’s wishes with regard to treatment options is important in preventing over-treatment.

It is important for ICUs and general wards to cooperate well, since there is a mutual dependency for optimal patient flow between the different departments. Interventions that improve the understanding and cooperation between these wards may help mitigate ethical problems.
